# Identification of Keystone Plant Species for Avian Foraging and Nesting in Beijing’s Forest Ecosystems: Implications for Urban Forest Bird Conservation

**DOI:** 10.3390/ani15152271

**Published:** 2025-08-04

**Authors:** Lele Lin, Yongjian Zhao, Chao Yuan, Yushu Zhang, Siyu Qiu, Jixin Cao

**Affiliations:** 1 Ecology and Nature Conservation Institute, Chinese Academy of Forestry, Beijing 100091, China; linlele@caf.ac.cn; 2 Beijing Xishan Experiment Forest Center, Beijing 100093, China; 15642070604@163.com; 3 Beijing Key Laboratory of Ecological Function Assessment and Regulation Technology of Green Space, Beijing Academy of Forestry and Landscape Architecture, Beijing 100102, China; ychy512@126.com (C.Y.); zys@yllhj.beijing.gov.cn (Y.Z.)

**Keywords:** bird conservation, urban forest, keystone species

## Abstract

Urban wildlife conservation is emerging as a critical component of sustainable city ecosystems. Rather than simply increasing tree abundance or species richness, conservation management should focus on key species. In this research, Xishan Forest Park in Beijing was chosen as a case study. Our aim was to identify keystone taxa critical for avian foraging and nesting during the breeding season. We performed a network analysis linking bird species, their diets, and nest plants. Dietary components were detected using DNA metabarcoding conducted with avian fecal samples. Nest plants were identified via transect surveys. Two indices of the network, degree and weighted mean degree, were calculated to evaluate the importance of the dietary and nest plant species. We identified 13 bird host species from 107 fecal samples and 14 bird species from 107 nest observations. Based on the degree indices, fruit trees *Morus* and *Prunus* were detected as key food sources, exhibiting both the highest degree (degree = 9, 9) and weighted mean degree (lnwMD = 5.21, 4.63). *Robinia pseudoacacia* provided predominant nesting sites, with a predominant degree of 7. A few taxa, such as *Styphnolobium japonicum* and *Rhamnus parvifolia*, served dual ecological significance as both essential food sources and nesting substrates. Scrublands, as a unique habitat type, provided nesting sites and food for small-bodied birds. Therefore, targeted management interventions are recommended to sustain or enhance these keystone resource species and to maintain the multi-layered vertical vegetation structure to preserve the diverse habitats of birds.

## Simple Summary

With accelerating urbanization, the urban ecosystem and wildlife conservation benefit expanding populations. Identifying keystone species for bird foraging and nesting enables targeted bird conservation in urban forests. By analyzing the associations of birds with their diets and nest plants in Beijing’s Xishan Forest Park during the breeding season, our study revealed the keystone dietary tree genera (*Morus* and *Prunus*) sustaining food webs and critical nest substrate species (*Robinia pseudoacacia*). The key food resources varied, along with phenological shifts such as fruit phases. Scrublands served as a unique habitat for small-bodied birds, providing nesting sites and food. These findings suggest management measures to sustain or enhance these keystone resource species and the maintenance of vertical vegetation stratification.

## 1. Introduction

Globally, the urban population has surpassed the rural population, and urbanization continues to advance [1]. This trend has amplified the importance of urban nature for the expanding population. Therefore, urban wildlife conservation is emerging as a critical component of sustainable city ecosystems [2,3]. Birds hold great ecological, economic, and cultural importance and are considered excellent indicators of ecosystem conditions because of their sensitive response to environmental change [4].

Both bird species richness and abundance are positively related to vegetation cover [5]. Secondary and planted vegetation provide suitable habitats for birds in urban and peri-urban areas. It is common for policymakers to preserve woodlots and plant trees to enhance bird diversity and resilience in these areas. However, rather than simply increasing tree abundance or species richness, conservation management should focus on key tree species [6]. These are often functionally important species in shaping avian survival and, therefore, determining community avian assemblage [7], e.g., bird-preferred nesting plants that could enhance nesting success and plants providing fruits/seeds or supporting invertebrate taxa as important components of bird diets. [8,9]. Thus, management measures involving these key taxa are likely to be most beneficial.

Reproductive success fundamentally determines population viability. Nesting is a crucial part of the avian reproductive process, where nests serve multiple purposes, including protection from predators and providing suitable environments for the developing eggs and offspring [10]. Certain tree species are more valuable for nesting than others because of their structural features, food sources, protection from predators and harsh weather conditions, etc. [11,12,13]. By analyzing the nest site selections of birds, previous studies have identified key nesting species during the breeding season. For instance, Mezquida analyzed the nest site preferences of five bird species breeding in southern South America and revealed three primary nesting plant species [8]. Brightsmith studied nesting records for 15 parrot species in southeastern Peru and identified two keystone plant resources used by nesting parrots [14]. Food availability is another widely accepted decisive factor for reproductive success [15]. In a certain community, a small arrangement of plant species can support considerably higher bird diversity than others. Some fruit/seed-bearing plants have been found to be keystone food sources for frugivores and granivores [16,17]. Some species provide foraging opportunities for insectivores [7]. Although many insect herbivores may be host-specific, a single host may support many different herbivores and thus support a much higher bird diversity [7].

The identification of key species was commonly based on studying their position in the network of interspecific interactions, in that the importance of a species may largely be the consequence of its rich interaction structure with birds [18,19]. For example, Messeder et al. constructed a plant–bird interaction network consisting of 373 genera and identified 6 genera as keystone resources for frugivores across the Neotropics [16]. Díaz et al. analyzed the bird–food interaction networks in Peru and revealed that nectar and fruits were the key food resources instead of insects, fish, and others [17]. In avian diet analysis, a combination of high-throughput sequencing and DNA metabarcoding across multiple taxa within a mixed sample has been broadly used as a powerful method [20]. Several DNA fragments have been used as standard barcodes for identifying different taxonomic groups in diets, e.g., *rbc*L and *mat*K for plant component identification and 12S rRNA and COI for animal component identification [21,22,23,24].

**Figure 1:**
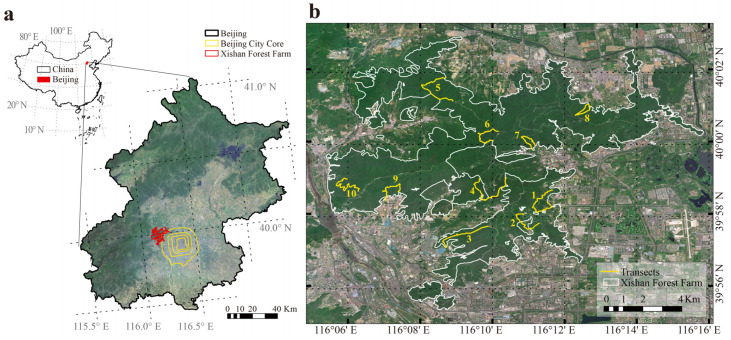
The location of Xishan Forest Park in Beijing, China (**a**), and distribution of transects in this study (**b**).

In this study, we selected Xishan Forest Park as the research area, which is in Beijing, one of the world’s premier megacities [1]. Located in Beijing’s western hilly area, the park represents the closest forest park to the urban core and sustains notably high avian diversity (Figure 1). In the management of its secondary and plantation forests, more proactive interventions can be implemented to enhance avian habitat quality and maintain bird diversity. We hypothesized that not all species within the community contribute equally to maintaining avian diversity. Certain key species exert more significant impacts. Therefore, this study intended to identify keystone taxa critical for avian foraging and nesting in Beijing Xishan Forest Park, thereby providing implications for targeted afforestation and silvicultural practices. The identification will be conducted through an interspecific network analysis of birds and their diets based on DNA metabarcoding and their nest plants based on a transect survey.

## 2. Materials and Methods

### 2.1. Transect Survey and Sample Collection

The study area was the closest forest park to the urban core of Beijing: Xishan Forest Park. The park covers an area of 57.68 km^2^, with the highest elevation reaching 798 m. The park has a warm, temperate, continental monsoon climate, with an annual average temperature of 9–11 °C and an average annual precipitation of 660 mm. The dominant forest species in the park include *Platycladus orientalis*, *Pinus tabuliformis*, *Acer truncatum*, *Quercus variabilis*, *Robinia pseudoacacia*, and *Cotinus coggygria*. A previous survey recorded a total of 101 bird species in the Xishan Forest Park [25].

To collect avian nest information and fecal samples, we conducted a transect survey in the park during the breeding season of 2024 from 4 June to 25 June. To ensure the transects covered various bird habitats, we established the survey transects based on the distribution of the vegetation types and dominant tree species within the study area. A total of 10 transect lines were set, each line ranging from 2 to 3.5 km in length (Figure 1). The transects covered deciduous forests, coniferous forests, mixed forests, and shrubs, representing communities with different dominant species, including *Ouercus variabilis*, *Acer truncatum*, *Koelreuteria paniculata*, *Robinia pseudoacacia*, *Pinus tabuliformis*, *Platycladus orientalis*, *Ailanthus altissima*, *Ulmus pumila*, *Rhamnus parvifolia*, *Celtis bungeana*, *Forsythia suspensa*, *Cotinus coggygria*, and *Vitex negundo*.

Bird nests were surveyed by direct observation within a 100 m buffer on either side of the transect lines. Information for each nest was recorded, including bird species, nest type, and nesting tree species. Tools used for observation included binoculars (Kowa BD 8 × 32, Kowa Company, Ltd., Nagoya, Japan) and a camera (Nikon Z8 400 mm lens, Nikon Corporation, Ayutthaya, Thailand). When breeding birds were present in the nests, the nest owners were identified through direct observation. If no breeding birds were observed in the nest, the nest owners were identified based on the nest’s location, shape, and construction materials. The nesting birds were identified according to the surveyors’ experience and references, including online databases [26,27], regional avifauna, field guides, and published papers [28,29,30].

Fresh bird feces were collected along the transects through random encounters, using disposable sterile gloves. Samples were selected based on their condition to ensure their freshness. To avoid repeated sampling of the same individual, we collected feces with intervals of more than five meters. Once collected, samples were immediately placed in a portable refrigerated box at 4 °C, subsequently transported to the laboratory within 10 h, and frozen at −80 °C before use.

### 2.2. DNA Extraction and Amplification

Total DNA was extracted from the fecal samples employing the E.Z.N.A. Stool DNA Kit (Omega Bio-tek, Norcross, GA, USA). The extracted DNA’s quality was evaluated through 1% agarose gel electrophoresis [31]. The plant chloroplast fragment *rbc*L gene was used to infer the plant components of the birds’ diets, and the mitochondrial fragment COI gene was used to infer the animal components [32,33]. The primer sequences and PCR protocol details for the amplification of these fragments are presented in Tables S1–S3. The PCR products were tested via 1% agarose gel electrophoresis and subsequently purified using the Agencourt AMPure XP kit (Beckman Coulter Life Sciences, Indianapolis, IN, USA).

### 2.3. Sequencing and Data Processing

High-throughput sequencing of the two DNA fragments was performed on a Miseq PE300/250 platform (Illumina, San Diego, CA, USA). Image analysis, base calling, and error estimation were conducted utilizing Illumina Analysis Pipeline v. 2.6. The raw sequencing data were subjected to quality filtering, which entailed the exclusion of sequences that were shorter than 230 bp, contained ambiguous bases, had a quality score of 20 or less, or did not have an exact match to the primer sequences and barcode tags. The reads that met the quality criteria were then clustered into operational taxonomic units (OTUs) at a 97% similarity threshold using the UPARSE algorithm in Vsearch version 2.7.1 [34]. Taxonomic classification of the sequences was performed with the BLAST tool (2.16.0) against the NCBI databases (National Center for Biotechnology Information [NCBI], Bethesda, MD, USA). For further analyses, only OTUs with an absolute abundance of more than 100 reads were included [35,36].

### 2.4. Data Analysis

Based on the OTU taxonomic classification results, the correspondence between the dietary components and avian taxa of each sample was identified. The most abundant avian taxon revealed by the COI sequence in each sample was considered the host [22]. To identify the key taxa providing food or nesting sites for birds during the breeding season, we constructed a network linking bird species (nodes) to their dietary and nesting flora and fauna taxa [37]. The networks were visualized by Gephi 0.10.1 [38]. Only edges with abundance exceeding 100 were incorporated into the network. We employed two indices to evaluate the importance of the plant nodes in the network, degree and abundance weighted mean degree (wMD). The degree of a node is the number of links (edges) connecting the node to the rest of the nodes in the network [37,39]. The wMD of a plant node is described by the following equation:

(1) $$wMD=\frac{\sum_{i=1}^{n}bs_{i}}{\sum_{i=1}^{n}b}$$

where *b* is the absolute abundance of the dietary taxa or nesting plants; *s_i_* is the abundance of the link between the bird species *i* and other nodes; *n* is the degree (amount of links) of the nodes representing dietary taxa or nesting plants [40]. Only edges with absolute abundance above 100 were included in the network. For the nodes of nesting plants, *b* and *s_i_* were calculated based on the nest amounts.

To analyze the preferences of birds for food plants and nesting plants, we calculated two indices: the food preference index (FP) and the nesting preference index (NP). The FP of a dietary plant genus was calculated as the ratio of its relative abundance in all dietary plants to its relative abundance in the study area’s plant community, and the NP of a nesting plant genus was calculated as the ratio of its relative abundance in all nesting plants to its relative abundance in the study area’s plant community. The detailed calculation methods are shown in Table S4.

To further investigate the temporal dynamics and species-specific variations in key food resource utilization, we performed non-metric multi-dimensional scaling (NMDS) based on Bray–Curtis dissimilarity with the R package “vegan” (v. 2.6-4) and linear discriminant analysis effective size (LEfSe) tests on the Galaxy online platform (http://huttenhower.sph.harvard.edu/galaxy/, accessed on 3 December 2024) [23,41,42,43].

## 3. Results

### 3.1. Diet Structure

**Figure 2:**
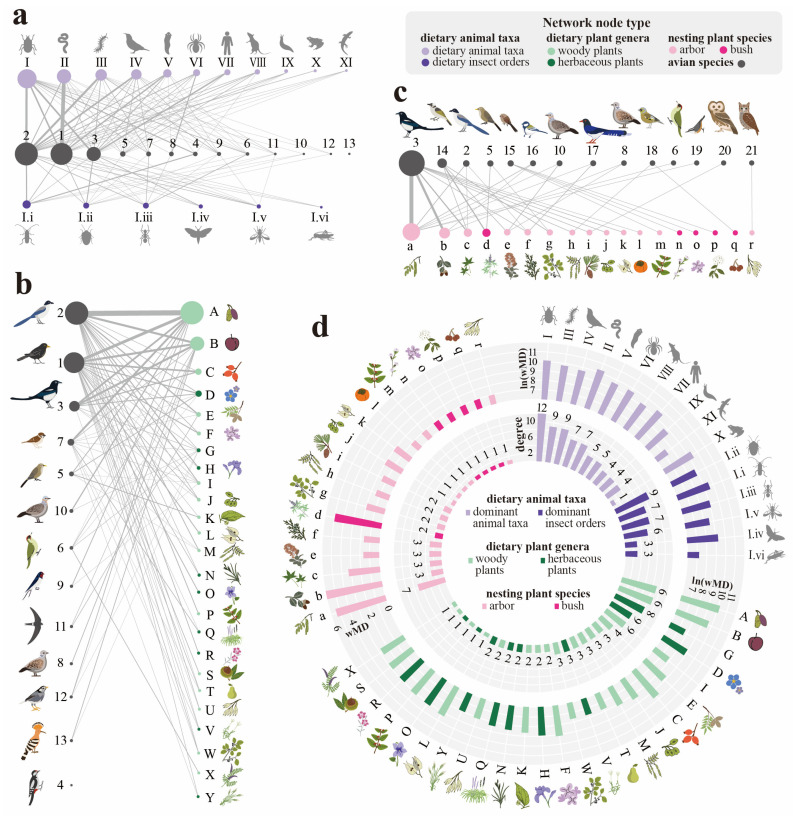
(**a**–**c**) Networks linking avian species to their dietary animal taxa (**a**), dietary plant genera (**b**), and nest-plant species (**c**). The size of the nodes is proportional to the abundance of each taxon, and so is the edge width to the link abundance. Only edges with abundance exceeding 100 were included in the network. (**d**) Degree and weighted mean degree (wMD) of food source taxa and nest-plants (avian species: 1 *Turdus mandarinus*, 2 *Cyanopica cyanus*, 3 *Pica pica*, 4 *Dendrocopos major*, 5 *Garrulax davidi*, 6 *Picus canus*, 7 *Passer montanus*, 8 *Streptopelia orientalis*, 9 *Hirundo rustica*, 10 *Spilopelia chinensis*, 11 *Apus pacificus*, 12 *Spodiopsar cineraceus*, 13 *Upupa epops*, 14 *Pycononotus sinensis*, 15 *Sinosuthora webbiana*, 16 *Parus minor*, 17 *Urocissa erythroryncha*, 18 *Chloris sinica*, 19 *Sitta villosa*, 20 *Strix nivicolum*, and 21 *Otus sunia*; dietary animal taxa: I Insecta, II Annelida, III other Arthropoda, IV Aves, V other invertebrates, VI Arachnidam, VII Hominidae, VIII Rodentia, IX Mollusca, X Amphibia, XI other vertebrates, I.i Coleoptera, I.ii Hemiptera, I.iii Hymenoptera, I.iv Lepidoptera, I.v Diptera, and I.vi other insects; dietary plant genera: A *Morus*, B *Prunus*, C *Rosa*, D *Cynoglossum*, E *Carya*, F *Syringa*, G other herbaceous plants, H *Iris*, I other woody plants, J *Ulmus*, K *Pteroceltis*, L *Koelreuteria*, M *Styphnolobium*, N *Carex*, O *Viola*, P *Lonicera*, Q *Liriope*, R *Dianthus*, S *Castanea*, T *Pyrus*, U *Ailanthus*, V *Digitaria*, W *Rhamnus*, X *Amorpha*, and Y *Triticum*; nest-plant species: a *Robinia pseudoacacia*, b *Quercus variabilis*, c *Acer truncatum*, d *Vitex negundo*, e *Cotinus coggygria*, f *Platycladus orientalis*, g *Rhamnus parvifolia*, h *Styphnolobium japonicum*, i *Pinus tabuliformis*, j *Ulmus pumila*, k *Koelreuteria paniculata*, l *Diospyros lotus*, m *Lonicera maackii*, n *Pertya dioica*, o *Syringa* spp., p *Spiraea* spp., q *Grewia biloba*, and r *Ailanthus altissima*).

A total of 107 fresh fecal samples were collected. Of all the 107 samples, 91 samples were successfully amplified and acquired COI sequences, and 97 samples successfully obtained *rbcL* sequences. Based on the COI taxonomic classification, 13 host bird species belonging to 10 families of five orders were identified, accounting for 12.87% of the 101 total recorded bird species in Xishan Forest Park. Eight of the identified host bird species were omnivorous species (Table S5; Figure 2a). The most abundant avian families were Corvidae and Turdidae, accounting for proportions of 48.35% and 28.26%, respectively. A total of 265 unique animal (Metazoa) OTUs were identified in the diets, belonging to 12 phyla (Figure S1). Invertebrates constitute the primary dietary component (75.89%). At the phylum level, Arthropoda demonstrated absolute dominance in the abundance (46.52%), followed by the phylum Annelida (18.12%). At the class level, Insecta was the most abundant animal food source (25.56%). At the order level, the five most abundant insect orders (Coleoptera, Hemiptera, Hymenoptera, Lepidoptera, and Diptera) accounted for 98% of the total class. The vertebrate components primarily consisted of birds, amphibians, and rodents. Notably, birds constituted the highest abundance (12.32%) of vertebrates, and human sequences were detected in some samples. Among the bird prey, the species with the highest abundance were *Garuulax davidi*, *Passer montanus*, and *Cyanopica cyanus* (Figure S2).

Based on the *rbc*L taxonomic classification, a total of 539 unique plant sequences (OTUs) were identified in the avian diets, belonging to 210 genera in 83 families. The abundance of different genera in the diets was not uniformly distributed but highly skewed to a limited number of dominant species (Figure 2b and Figure S3). The cumulative average relative abundance of the top 15 genera reached 70.99%. In particular, the top two genera, *Morus* and *Prunus*, exhibited prominent dominance with average relative abundances of 25.44% and 13.74%, respectively. Woody genera constituted the majority of dietary plants, especially fruit trees. Among the 44 genera with an average relative abundance exceeding 0.1%, 25 genera were woody plant genera, accounting for a cumulative average relative abundance of 82.05% (8 fruit tree genera accounting for 64.00%), while 19 genera were herbaceous ones, with a cumulative relative abundance of 17.95%. According to the results of the feeding preference index calculations, four genera exhibited feeding preference, including *Morus*, *Styphnolobium*, *Prunus*, and *Rhamnus*, with feeding preference indices of 4.89, 1.45, 0.45, and 0.15, respectively (Figure S4). The UpSet plot results of the overlap analysis indicate that *Cyanopica cyanus*, *Turdus mandarinus*, and *Pica pica* had the highest dietary overlap with other species (Figure S5).

### 3.2. Feeding and Nesting Relationship Networks

A total of 147 bird nests were surveyed, of which 116 could be attributed to identifiable bird species. Among these nests, nine were located on artificial structures or rocks, and the remaining 107 were found in vegetation. These vegetation nests belonged to 14 distinct bird species of four orders and 10 families (Table S5, Figure 2c). The top two avian species exhibiting the highest observed nest abundance were *Pica pica* (58.88%) and *Pycnonotus sinensis* (13.08%). Four plant species supported significantly greater numbers of nests compared to other vegetation. Broadleaf trees demonstrated absolute dominance over conifers and shrubs as preferred nesting substrates. The four dominant nest-plant species, ranked by nest abundance, were *Robinia pseudoacacia* (35.51%), *Quercus variabilis* (15.89%), *Acer truncatum* (9.35%), and *Vitex negundo* (9.35%). As preferred nesting substrates, deciduous trees demonstrated absolute dominance (78.50%) over coniferous trees (7.48%) and shrubs (14.02%). According to the results of the nesting preference index calculations, eight genera exhibited nesting preference, the top four including *Styphnolobium*, *Rhamnus*, *Diospyros*, and *Quercus*, with feeding preference indices of 2.34, 1.82, 1.24, and 0.89, respectively (Figure S3).

The ecological network (Figure 2a–c) visualized the trophic and nesting interactions between avian species and other biological taxa within the community. The bird–dietary animal network contained 99 trophic links connecting 13 bird species with 17 prey taxa, including six dominant orders from the class Insecta (Figure 2a). The bird–dietary plant network featured 80 trophic associations between 13 avian species and 25 dominant plant genera (Figure 2b). The nesting network consisted of 107 ecological linkages involving 14 bird species and their 18 nest-plant species (Figure 2c).

Node degree quantified the number of avian species consuming or nesting on each plant/animal taxon, while weighted mean degree (wMD) incorporated both edge frequency and node abundance to measure interaction strength (Figure 2d). Among animal nodes, insects supported the greatest diversity of avian consumers, and Annelids exhibited the highest wMD values, indicating the strongest trophic importance. *Morus* and *Prunus* were identified as the most important food resources by both degree and wMD. Interestingly, certain genera, such as *Rosa* and *Syringa*, exhibited a distinct interaction pattern characterized by a relatively low degree but a high wMD, potentially indicating specialized avian foraging preferences or disproportionate ecological importance for particular bird species. Key nesting substrate plants were frequently dominant tree species in the community, such as *Robinia pseudoacacia*, *Acer truncatum*, and *Cotinus coggygria*. Some species, such as *Vitex negundo*, provided nesting sites for only a minimal number of bird species but maintained an exceptionally high wMD, suggesting strong preference or ecological dependence among these nesters. Notably, only limited overlap occurred between primary dietary plant species and critical nesting tree species. This highlighted the dual ecological significance of the few overlap taxa, such as *Styphnolobium japonicum* and *Rhamnus parvifolia*, which serve both as essential food sources and nesting substrates.

### 3.3. Temporal and Species-Specific Dietary Variations

**Figure 3:**
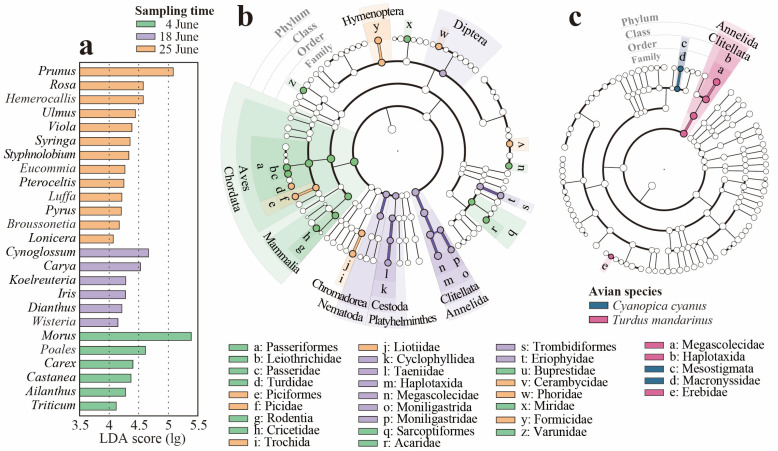
Differentially abundant dietary taxa based on linear discriminant analysis effective size (LEfSe). (**a**) Plant genera differentially abundant across the sampling times (LDA cut-off value = 4.0, *p* < 0.05); (**b**) animal taxa differentially abundant across the sampling times (LDA cut-off value = 4.0, *p* < 0.05); (**c**) animal taxa differentially abundant in avian species (LDA cut-off value = 3.0, *p* < 0.05, only species with sample sizes exceeding 3 were included in the analysis).

The NMDS analysis showed no significant differences in dietary plant composition among avian species, but potentially different components across sampling times may exist (Figure S6). The Sankey diagram also revealed notable shifts in consumption of dominant genera across times (Figure S7). To further confirm the differential components, LEfSe analysis was conducted, and 25 genera that were differently abundant in avian diets across time were identified (Figure 3a). For instance, the genus *Morus* was most abundant at the beginning of the month and less prevalent at the end. The genus *Prunus* was less common at the beginning of the month and most abundant at the month’s end.

In the dietary animal taxa, both the NMDS results and the Sankey diagram suggested that potential differential components may exist among bird species and across sampling times (Figures S8 and S9). Thus, LEfSe analysis was conducted and identified temporally differentially abundant taxa: Aves and Mammalia were more abundant in the early month; Diptera peaked in abundance during mid-June; and Hymenoptera was concentrated at the end of the month (Figure 3b). In avian species with a sample size exceeding three, the LEfSe analysis revealed significantly higher consumption of the phylum Annelida by *Cyanopica cyanus* and of the order Mesostigmata by *Turdus merula*, while no significant dietary differences were detected in *Pica pica* (Figure 3c).

## 4. Discussion

### 4.1. Avian Foraging Preference and Flexibility

Food availability is a primary driver of avian reproductive success [15,44,45]. For suburban birds, the anthropogenic environment provides additional food resources, such as cultivated fruits, crops, and food waste [46,47]. In this study, the consumption of cultivated plants such as *Carya*, *Triticum*, and *Liriope* was detected, and human-derived components were also found in avian diets, notably prevalent in *Pica pica* (Figure 2a,b, Figures S1 and S3). This supplementary food availability shapes avian reproduction and bird community structures. Studies have shown that cultivated plants can provide increased foraging opportunities and prolong the avian breeding duration [47], and the additional anthropogenic food supply could increase reproductive performance for birds with a broader diet [48,49]. Meanwhile, birds with a higher tolerance for human disturbance and more generalized diets are more inclined to consume those anthropogenic foods, e.g., *Pica pica* and *Passer domesticus* [15,50]. Therefore, urbanization tends to select for omnivorous species with a high tolerance for human disturbance [51]. Such species thus commonly exhibit high population densities within cities, as observed in this study (Table S5).

To meet the increasing nutritional demands during breeding and to guarantee reproduction success in a changing environment, birds exhibit dietary flexibility in response to the phenology of dietary taxa [52,53]. Urban birds have been indicated to be more plastic in response to the heterogeneous environment than their non-urban neighbors [54]. Our results detected temporal variations in the avian diets. The consumption of the two most abundant genera, *Morus* and *Prunus*, peaked during the early and late phases of the month, respectively (Figure 3a). This may reflect the birds’ foraging variations in response to differences in fruit hanging duration during the plants’ ripening periods [55]. The feeding tendencies of birds have been proved to be influenced by the hanging status of fruit, with fresh and plump fruits being more attractive, such that poorly maintained fruits (such as *Morus* and *Prunus*) are only eaten at the early stage of fruit ripening [56]. This might explain why the avian diets shifted within such a short period of time. Meanwhile, the abundance of certain phytophagous insects appears to be synchronized with their host plants, such as *Morus*, and Miridae insects (Figure 3b). This may occur because insects appearing on plants bearing ripe fruits face a higher risk of predation [57]. It should be pointed out that the relatively short sampling period in this study limits its ability to reflect seasonal phenological changes. Therefore, the implications of this study lie in the shaping of bird foraging behavior by short-term variations in food availability.

Apart from food availability, diets also reflect food preference [58]. Food preference is shaped by the combined effects of morphological adaptations, predation risk, spatial distribution of food resources, and trophic trade-offs. [59,60,61,62]. In this study, no significant difference in dietary plants was found among avian species (Figure S6). This may be attributed to the broader dietary range of the dominant species overlapping with and encompassing that of their minor counterparts (Figure S5). From the perspective of guilds, most of the bird species in this study were omnivores, and the dominant species fed on especially broader diets. In terms of morphological matching, large predators can feed on foods of different sizes, whereas smaller predators are more restricted to small foods [63]. Dominant avian species (such as *Cyanopica cyanus*, *Pica pica*, and *Turdus mandarinus*) demonstrated relatively large body sizes and broader dietary ranges. Interspecific variations in the consumptions of dietary animals in this study indicated food preference differentials. Phylogenetically similar species often show a similar preference due to morphological trait matching [64]. As was observed in this study, the primary dietary taxon for *Turdus mandarinus* was Annelida (Figure 2a and Figure S9), possibly because of the preference of their nestlings for protein-rich and digestible earthworms [65]. Conversely, insects were the primary food sources for woodpeckers (*Dendrocopos major* and *Picus canus*) (Figure 2a and Figure S9), probably driven by their arboreal adaptations and less common ground foraging.

### 4.2. Key Taxa and Implications for Forest Management

Based on the OTU taxonomic classification results, the avian diets contained typical forest pests in north China, such as *Aegosoma sinicum*, *Holotrichia oblita*, the *Apolygus* sp. of Insecta, and the *Aceria* sp. of Arachnida (Figure S1). The forest pest taxa constituted more than half of the abundance within the class Insecta. These indicated avian contributions to pest control and the importance of conserving bird diversity in forest management [66,67].

The high abundance of avian components in diets may reflect the presence of relatively intense nest predation. The enrichment of avian diets early in the month may correspond to the peak period of chick hatching in the breeding season (Figure 3b). Nest predation has been observed to be the primary source of nest losses [68]. Corvids are typical nest predators [69,70], as was observed in this study (Figure S2). This may pose risks to some rare bird species, in that low productivity resulting from high nest predation can be one of several potential causes of bird population declines [71]. For example, in this study, the abundance of *Garrulax davidi* was not the highest among the surveyed birds, but it was the most abundant among the bird prey (Figure S2). Therefore, we suggest that further research be conducted on nest predation in the study area to investigate its influence on prey species and avian diversity.

In terms of both foraging and nesting, deciduous trees were the taxa with the highest degrees (Figure 2d). Similar results have been found in previous studies on birds in surrounding forests [72,73,74]. The primary nesting tree species were mostly dominant species in the plant community, e.g., *Robinia*, *Quercus*, and *Acer* (Figure 2d and Figure S4). Among all the nesting trees in this study, *Robinia pseudoacacia* exhibited the highest degree. It was observed in this survey that large-diameter *R. pseudoacacia* provided the most cavity nesting sites. Currently, forest management often involves the regeneration and transformation of artificial *R. pseudoacacia* plantations due to the degradation of their ecological functions [75]. From the perspective of bird diversity conservation, this study shows that some large-diameter deciduous species or trees with cavities need to be retained to meet avian nesting needs. Fruit tree genera such as *Morus* and *Prunus* play a central role in the network as dietary plants. In forest management, these species should be considered key species, with efforts made to preserve their populations or to plant additional individuals.

Although not predominant, shrubs provide suitable nesting and foraging environments for small-sized birds. For example, in this study, shrubs composed of *Vitex negundo*, *Pertya dioica*, *Syringa* spp., *Spiraea* spp., and *Grewia biloba* serve as the primary nesting plants for the small-bodied bird *Sinosuthora webbiana* and also provide a considerable portion of the nesting plants for other small-bodied birds, such as *Garrulax davidi* and *Chloris sinica*. (Figure 2c). It has been indicated that avian body size has a significant impact on the choice of nesting tree species. Birds with large bodies prefer tall trees for sufficient support, while small-bodied birds favor shrubs or small trees [76]. Shrubs beneath forest canopies form a multi-layered vertical vegetation structure, which has been proven to provide birds with more diverse habitats and thereby maintain avian diversity [77,78].

## 5. Conclusions

Avian foraging exhibited phenological plasticity, dynamically tracking the availability of food resources. *Morus* and *Prunus* were key components as food sources, although they are not dominant species in the plant community. *Robinia pseudoacacia* was the key nesting plant for birds. Scrublands, as a unique habitat type, provided nesting sites and food for small-bodied birds. We suggest focusing on these keystone resource species and the multi-layered vertical vegetation structure in the conservation of bird diversity in the city forest.

## Data Availability

The dataset is available upon request from the authors.

## Supplementary Materials

The following supporting information can be downloaded at https://www.mdpi.com/article/10.3390/ani15152271/s1: Table S1: The primers used to infer avian diets. Table S2: The PCR system used for DNA amplification. Table S3: The PCR protocol used for DNA amplification. Table S4: The calculation method for the food preference (FP) and nesting preference (NP) indices. Table S5: The avian species surveyed in this study. Figure S1: Accumulated relative abundance of major dietary animal taxa. Figure S2: Sankey diagram illustrating quantitative relationship between avian prey and predators. Figure S3: Accumulated relative abundance of major dietary plant genera. Figure S4: Feeding and nesting preferences of birds for different plant genera. Figure S5: UpSet diagram illustrating overlap between different avian species. Figure S6: Non-metric multi-dimensional scaling (NMDS) of the dietary plants among different sampling times and avian species. Figure S7: Sankey diagram illustrating allocation of dietary plant genera across sampling times. Figure S8: Non-metric multi-dimensional scaling (NMDS) of the dietary animals among different sampling times and avian species. Figure S9: Sankey Diagram illustrating allocation of dietary animal taxa across sampling times and avian species.
