# Supplementary material for: Identification of Keystone Plant Species for Avian Foraging and Nesting in Beijing’s Forest Ecosystems: Implications for Urban Forest Bird Conservation
Source: Animals (Basel). 2025 Aug 4;15(15):2271. doi: 10.3390/ani15152271 (PMC12345470; doi:10.3390/ani15152271)
Supplement: Supplementary file 1 [file animals-15-02271-s001.zip › supplementary materials-animals-3703521.pdf]

Supplementary Materials

Table S1. The primers used to infer avian diets

Fragment	Primer sequence
*rbc*L2	CTTACCAGYCTTGATCGTTACAAAGG
	GTAAAATCAAGTCCACCRCG
COI	GGWACWGGWTGAACWGTWTAYCCYCC
	TAIACYTCIGGRTGICCRAARAAYCA

Table S2. The PCR system used for DNA amplification

Component	Volume
DNA sample	30 ng
Forward primer (5 uM)	1 μL
Reverse primer (5 uM)	1 μL
BSA (2 ng/μL)	3 μL
2×Taq Plus Master Mix	12.5 μL
ddH_2O	7.5-× μL
Total	25 μL

Table S3. The PCR protocol used for DNA amplification

DNA fragment	reaction protocol	
*rbc*L	94 ℃	5 min
	------------------------------	
	35 cycles:	
	94 ℃	30 s
	55 ℃	30 s
	72 ℃	60 s
	------------------------------	
	72 ℃	7 min
	4 ℃	end
COI	94 ℃	5 min
	------------------------------	
	45 cycles:	
	94 ℃	30 s
	52 ℃	30 s
	72 ℃	60 s
	------------------------------	
	72 ℃	10 min
	4 ℃	end

Table S4. The calculation method of food preference (FP) and the nesting preference (NP) indices

No.	Equation	Explanation
1	$FP = \ln\left(\frac{f_i}{c_i}\right)$	FP (feeding preference) of a dietary plant genus is calculated as the ratio of its relative abundance in all

		dietary plants (f_i) to its relative abundance in the study area's plant community (c_i).
2	$NP = \ln\left(\frac{n_i}{c_i}\right)$	*NP* (nesting preference) of a nesting plant genus is calculated as the ratio of its relative abundance in all dietary plants (n_i) to its relative abundance in the study area's plant community (c_i).
3	$c_i = \frac{\sum s_i^{dom}}{S}$	c_i is the relative abundance of genus *i* in the study area's plant community. To calculate the value of c_i, we obtained records of 1,867 bird observation sites from Xishan Forest Park and extracted dominant species of each site form the records. s_i^{dom} is calculated as the number of observation sites where the plant genus *i* is dominant species. *S* is the total number of points, with the value of 1,867 in this case. If a point has only one dominant species, the s_i^{dom} of the genus is assigned a value of 1. If a point has two dominant species, the s_i^{dom} of each genus is assigned a value of 0.5.

Table S5. The avian species identified in this study

Order	Family	Species	Residential type*
		Dietary survey	
Columbiformes	Columbidae	*珠颈斑鸠 Spilopelia chinensis*	R
Columbiformes	Columbidae	*山斑鸠 Streptopelia orientalis*	R
Caprimulgiformes	Apodidae	*白腰雨燕 Apus pacificus*	S, P
Bucerotiformes	Upupidae	*戴胜 Upupa epops*	S
Piciformes	Picidae	*大斑啄木鸟 Dendrocopos major*	R
Piciformes	Picidae	*灰头绿啄木鸟 Picus canus*	R
Passeriformes	Corvidae	*灰喜鹊 Cyanopica cyanus*	R
Passeriformes	Corvidae	*喜鹊 Pica pica*	R
Passeriformes	Hirundinidae	*家燕 Hirundo rustica*	S, P
Passeriformes	Leiothrichidae	*山噪鹛 Garrulax davidi*	R
Passeriformes	Sturnidae	*灰椋鸟 Spodiopsar cineraceus*	W, R
Passeriformes	Turdidae	*乌鸫 Turdus mandarinus*	R
Passeriformes	Fringillidae	*麻雀 Passer montanus*	R
		Nesting survey	
Columbiformes	Columbidae	*珠颈斑鸠 Spilopelia chinensis*	R
Columbiformes	Columbidae	*山斑鸠 Streptopelia orientalis*	R
Strigiformes	Strigidae	*红角鸮 Otus sunia*	S
Strigiformes	Strigidae	*灰林鸮 Strix nivicolum*	R
Piciformes	Picidae	*灰头绿啄木鸟 Picus canus*	R
Passeriformes	Corvidae	*红嘴蓝鹊 Urocissa erythroryncha*	R
Passeriformes	Corvidae	*灰喜鹊 Cyanopica cyanus*	R
Passeriformes	Corvidae	*喜鹊 Pica pica*	R

Passeriformes	Paridae	大山雀 *Parus minor*	R
Passeriformes	Pycnonotidae	白头鹎 *Pycnonotus sinensis*	R
Passeriformes	Leiothrichidae	山噪鹛 *Garrulax davidi*	R
Passeriformes	Sittidae	黑头鳾 *Sitta villosa*	R
Passeriformes	Paradoxornithidae	棕头鸦雀 *Sinosuthora webbiana*	R
Passeriformes	Fringillidae	金翅雀 *Chloris sinica*	R

*Residential type: R. Resident; S. Summer migrant; W. Winter migrant; P. Passage migrant

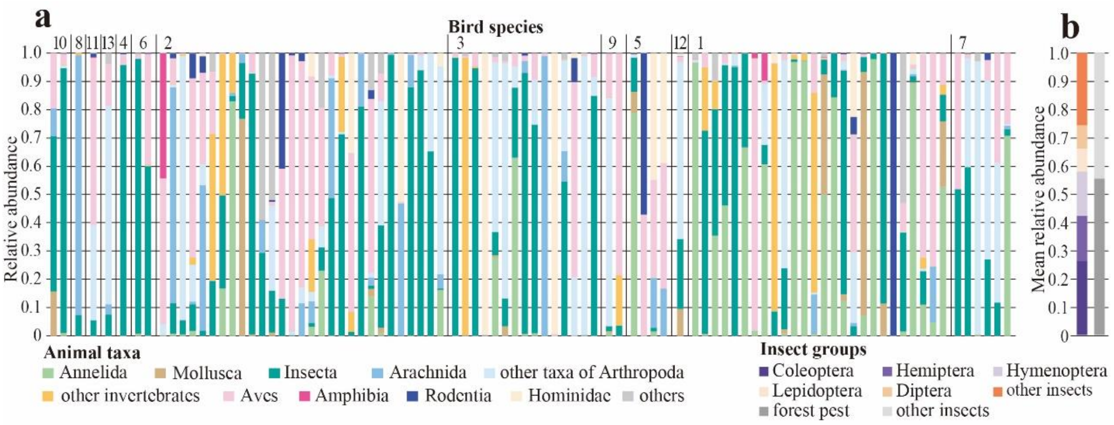

Figure S1. Accumulated relative abundance of major dietary animal taxa (a) and mean relative abundance of major groups in the class Insecta (b). (avian species: 1. *Turdus mandarinus*, 2. *Cyanopica cyanus*, 3. *Pica pica*, 4. *Dendrocopos major*, 5. *Garrulax davidi*, 6. *Picus canus*, 7. *Passer montanus*, 8. *Streptopelia orientalis*, 9. *Hirundo rustica*, 10. *Spilopelia chinensis*, 11. *Apus pacificus*, 12. *Spodiopsar cineraceus*, 13. *Upupa epops*)

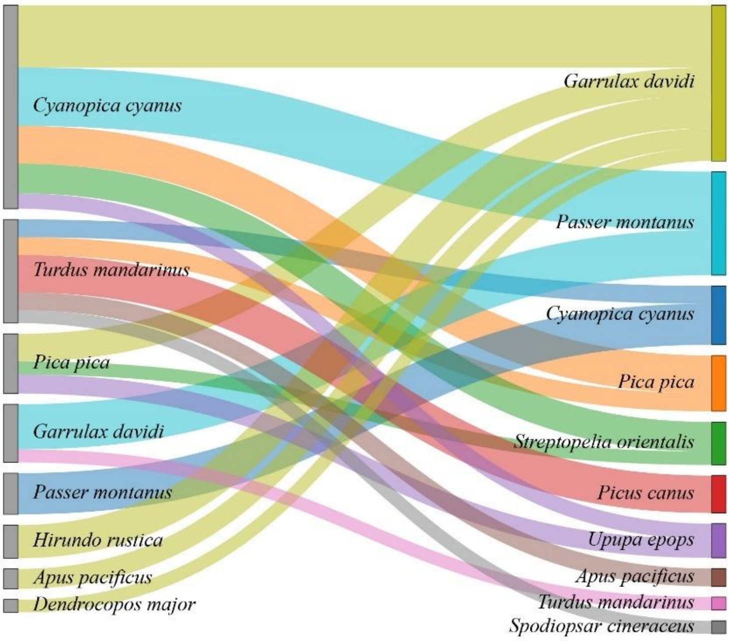

Figure S2. Sankey diagram illustrating quantitative relationship between avian prey (right) and predators (left).

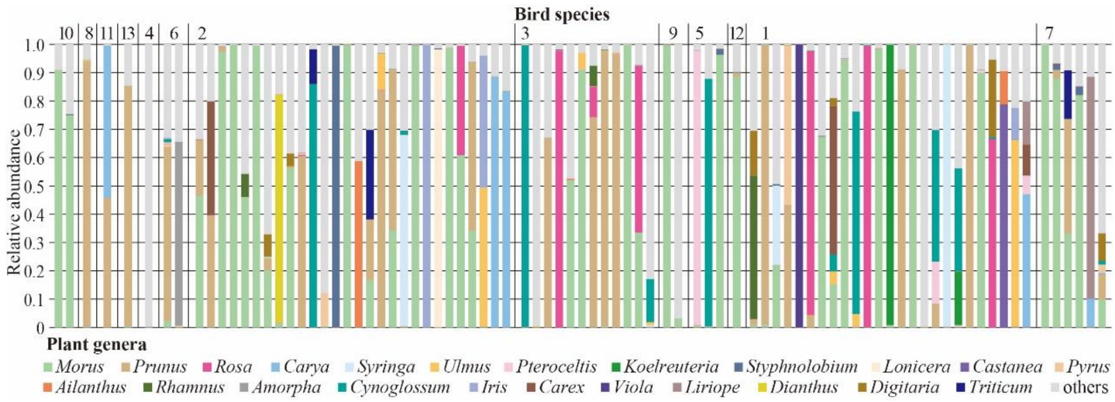

Figure S3. Accumulated relative abundance of major dietary plant genera (Only genera with accumulated average abundance > 75% were exhibited; avian species: 1. *Turdus mandarinus*, 2. *Cyanopica cyanus*, 3. *Pica pica*, 4. *Dendrocopos major*, 5. *Garrulax davidi*, 6. *Picus canus*, 7. *Passer montanus*, 8. *Streptopelia orientalis*, 9. *Hirundo rustica*, 10. *Spilopelia chinensis*, 11. *Apus pacificus*, 12. *Spodiopsar cineraceus*, 13. *Upupa epops*)

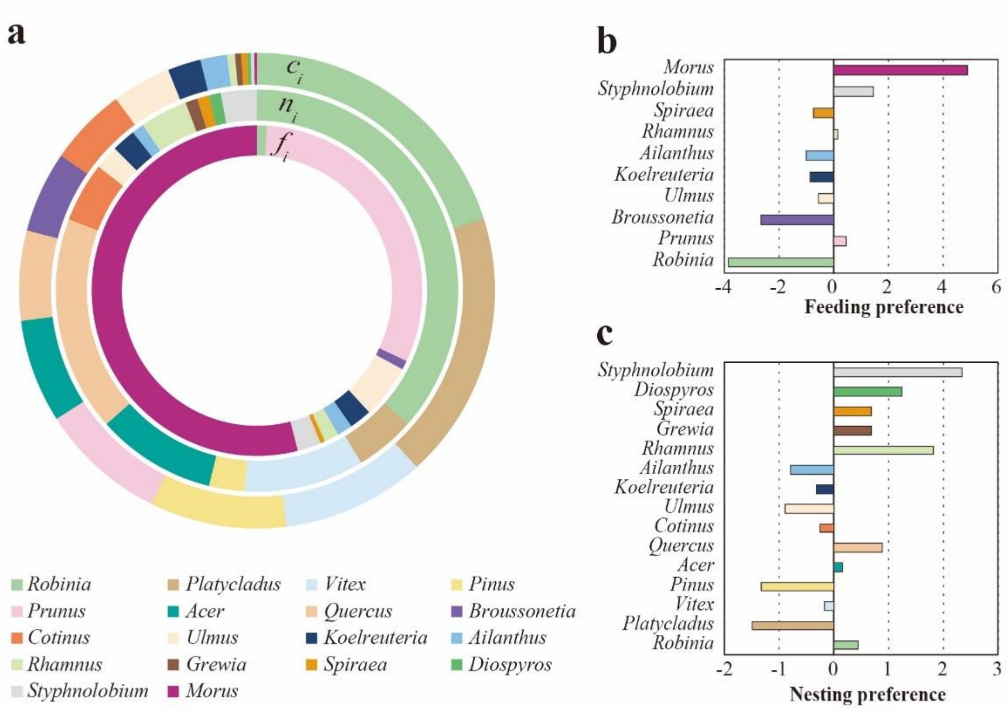

Figure S4. Feeding and nesting preferences of birds for different plant genera.
(Calculating methods of feeding and nesting preference indices was shown in Table S4.
a. c_i: the relative abundance of each genus in the study area's plant community; f_i: relative abundance of each genus in all dietary plants; n_i: relative abundance of each genus in all dietary plants.
b. feeding preference indices of main dietary plant genera.
c. nesting preference indices of main nesting plant genera.)

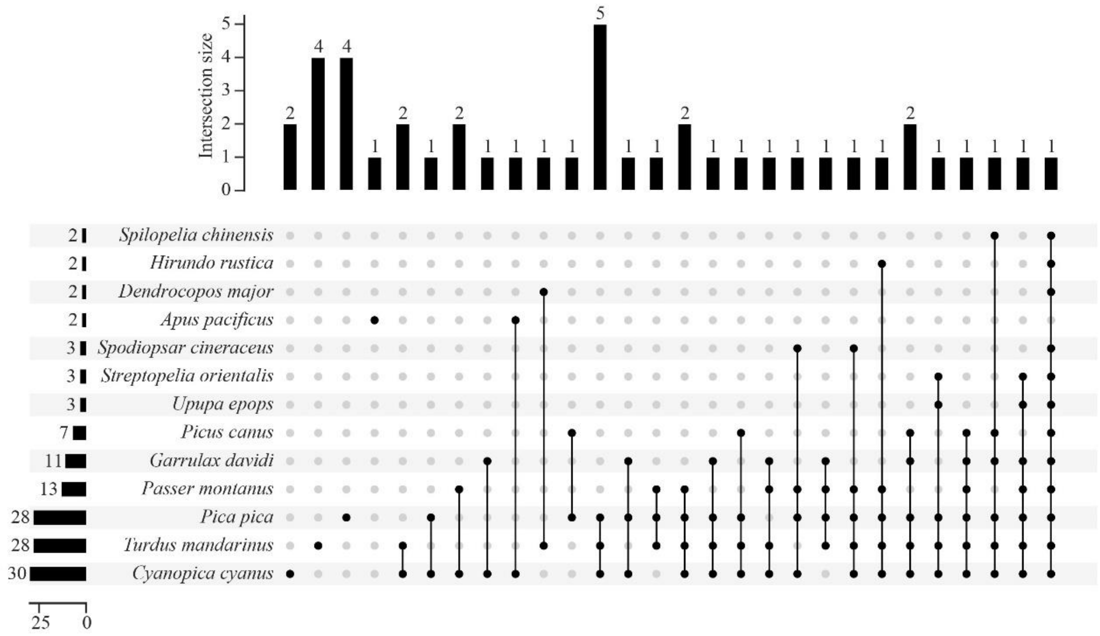

Figure S5. UpSet diagram illustrating overlap between different avian species based on 23 dietary plant genera and 16 dietary animal taxa (plant genera: *Morus*, *Prunus*, *Rosa*, *Cynoglossum*, *Carya*, *Syringa*, *Ulmus*, *Pteroceltis*, *Koelreuteria*, *Styphnolobium*, *Lonicera*, *Castanea*, *Pyrus*, *Ailanthus*, *Rhamnus*, *Amorpha*, *Iris*, *Carex*, *Viola*, *Liriope*, *Dianthus*, *Digitaria*, *Triticum*; animal taxa: Annelida, Mollusca, Insecta, Arachnida, other Arthropoda, other invertebrates, Aves, Amphibia, Human, Rodentia, other vertebrate, Coleoptera, Diptera, Hemiptera, Hymenoptera, Lepidoptera).

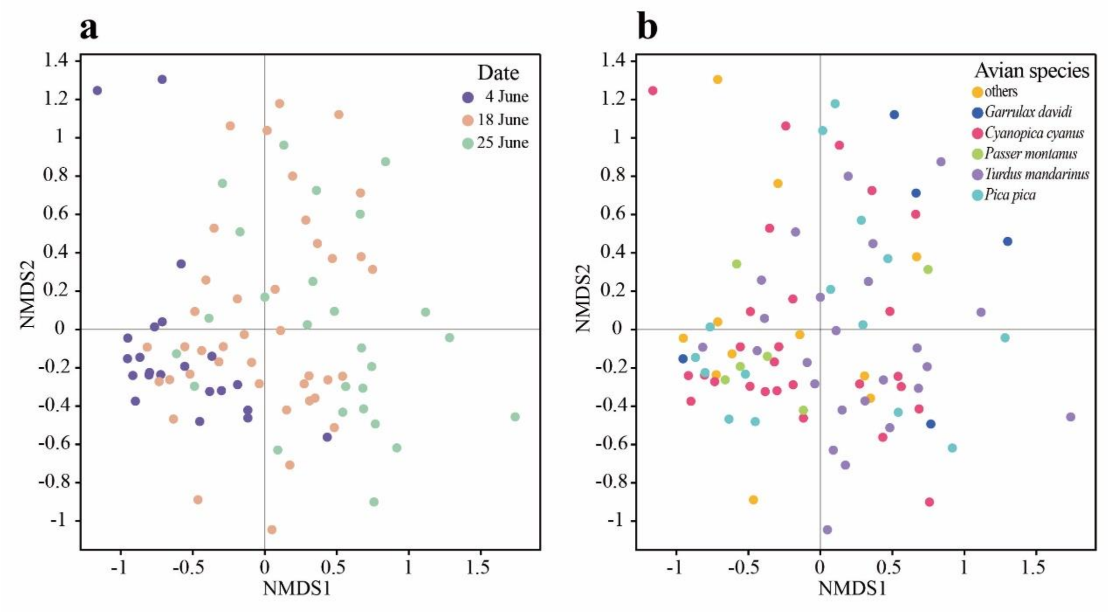

Figure S6. Non-metric multi-dimensional scaling (NMDS) of the dietary plants among different sampling times (a) and avian species (b).

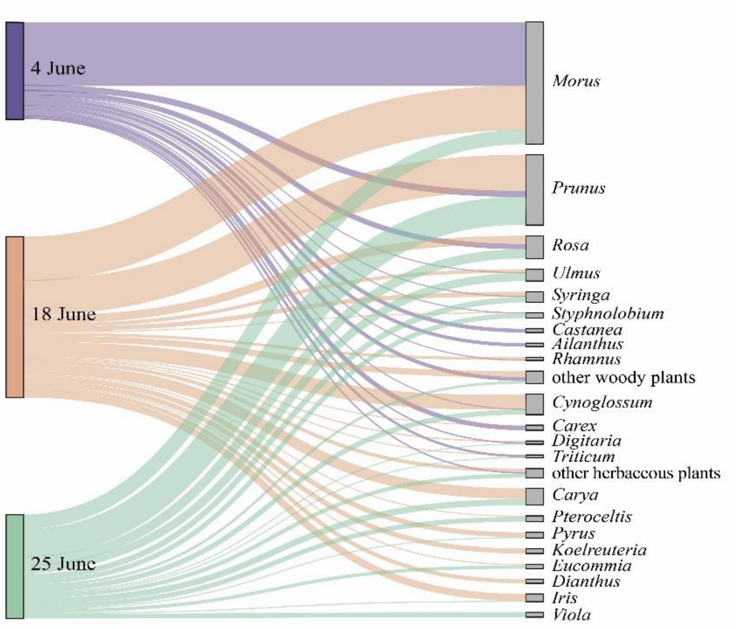

Figure S7. Sankey diagram illustrating allocation of dietary plant genera across sampling times.

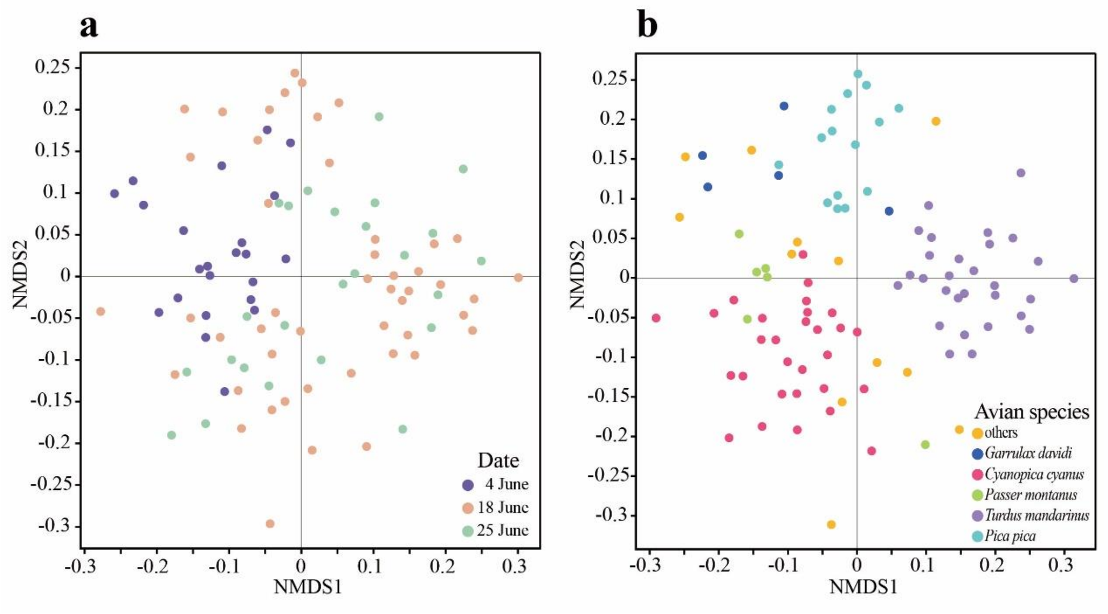

Figure S8. Non-metric multi-dimensional scaling (NMDS) of the dietary animals among different sampling times (a) and avian species (b).

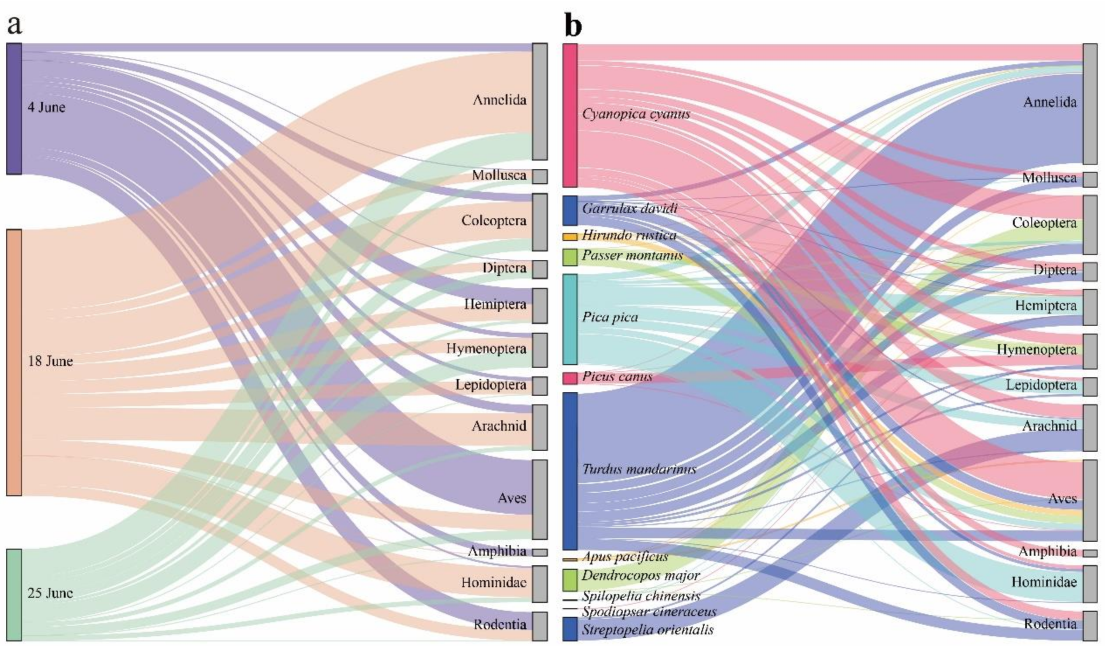

Figure S9. Sankey Diagram illustrating allocation of dietary animal taxa across sampling times (a) and avian species (b).
